# The E3 ubiquitin ligase NEDD4 is translationally upregulated and facilitates pancreatic cancer

**DOI:** 10.18632/oncotarget.15446

**Published:** 2017-02-17

**Authors:** Min Weng, Zhu-Lin Luo, Xiao-Ling Wu, Wei-Zheng Zeng

**Affiliations:** ^1^ Department of Gastroenterology, Chengdu Military General Hospital, Chengdu, Sichuan 610083, China; ^2^ Department of General Surgery, Chengdu Military General Hospital, Chengdu, Sichuan 610083, China

**Keywords:** pancreatic ductal adenocarcinoma, PDAC, NEDD4, PTEN, PI3K/AKT

## Abstract

**Aim:**

To determine the regulation and function of the neural precursor cell expressed developmentally down regulated protein 4 (NEDD4) in PDAC and to determine its dependency on phosphatase and tensin homolog (PTEN) and PI3K/AKT signaling.

**Methods:**

We investigated the expression of NEDD4 and the tumor suppressor PTEN in normal immortalized human pancreatic duct epithelial cell line and pancreatic adenocarcinoma (PDAC) cell lines. We further evaluated whether RNAi-mediated depletion of NEDD4 can attenuate PDAC cell proliferation and migration. We subsequently determined the crosstalk between NEDD4 expression and the PTEN/PI3K/AKT signaling pathway. Finally, we determined the mechanism behind differential NEDD4 protein expression in pancreatic cancer.

**Results:**

The expression of NEDD4 was heterogeneous in PDAC cells, but was significantly higher compared to normal pancreatic ductal epithelial cells. Analogically, PTEN was decreased in the PDAC cells. A combination of MTT assay, wound healing migration assay, and transwell invasion assays confirmed that depletion of *NEDD4* decreased the proliferation and migration ability of PDAC cells. Western blot and immunofluorescence results revealed that NEDD4 could affect PTEN/PI3K/AKT signaling pathway in PDAC cells. Polysomal profiling revealed that higher NEDD4 protein expression in PDAC cells was due to undefined mechanism involving translational activation.

**Conclusions:**

Our results reveal a novel mechanism of upregulation of NEDD4 expression in PDAC. Our findings indicate that NEDD4 potentially plays a critical role in activating the PI3K/AKT signaling pathway by negatively regulating PTEN levels in PDAC cells, which promotes pancreatic cancer cell proliferation and metastasis. Therefore, NEDD4 may be a potential therapeutic target in PDAC.

## INTRODUCTION

Pancreatic cancer causes an estimated 227,000 deaths per year worldwide [1–3]. Pancreatic ductal adenocarcinoma (PDAC) is the most common histological type of pancreatic cancer [1] presenting with a highly invasive and metastatic phenotype, which in turn is often responsible for treatment failure and an extremely poor clinical prognosis [3, 4]. Hence, it is important to identify prognostic and diagnostic markers that can be clinically implemented for optimized treatment strategies for pancreatic cancer patients.

The neural precursor cell-expressed developmentally down regulated gene 4 (*NEDD4*) was the founding member of the homologous to E6-AP carboxyl terminus (HECT)-type E3 ligases family [5]. Previous studies have shown that ubiquitination does play an important role in controlling the turnover rate, localization and activity of cellular proteins [6]. NEDD4 was initially identified as a critical protein in regulating neuronal function and plasticity in the brain [7]. Since NEDD4 itself is regulated by ubiquitination, phosphorylation and other post-translational modifications, further evidence described the involvement of aberrant NEDD4 expression in cells with damages in the regulatory ubiquitination process. These aberrations lead to malignant transformations, underlying the significance of NEDD4 in tumorigenesis and cancer development [8, 9].

*PTEN* (phosphatase and tensin homolog), a tumor suppressor gene with diverse functions in various tissues, is a negative regulator of the PI3K/AKT signaling pathway. It dephosphorylates PIP3 and thus interferes with AKT activation [10]. *PTEN* expression is down regulated in several cancer types, including pancreatic cancer [11–13]. Interestingly, recent evidences have suggested *NEDD4* as an oncoprotein, since it induces proteasomal degradation of the tumor suppressor PTEN [14–16].

NEDD4 overexpression and its role in cancer growth promotion (independently of PTEN and PI3K/AKT signaling) were shown in colorectal cancers, in prostate and bladder cancer, and indirectly in esophageal cancer. However, the function and interactions of NEDD4 and PTEN in pancreatic ductal adenocarcinoma (PDAC) still remains elusive. Hence, the objective of the current study was to investigate the regulation and function of NEDD4 in PDAC and to determine its dependency on PTEN and PI3K/AKT signaling.

## RESULTS

### NEDD4 and PTEN protein expressions in normal pancreatic ductal epithelium and PDAC cell lines

**Figure 1:**
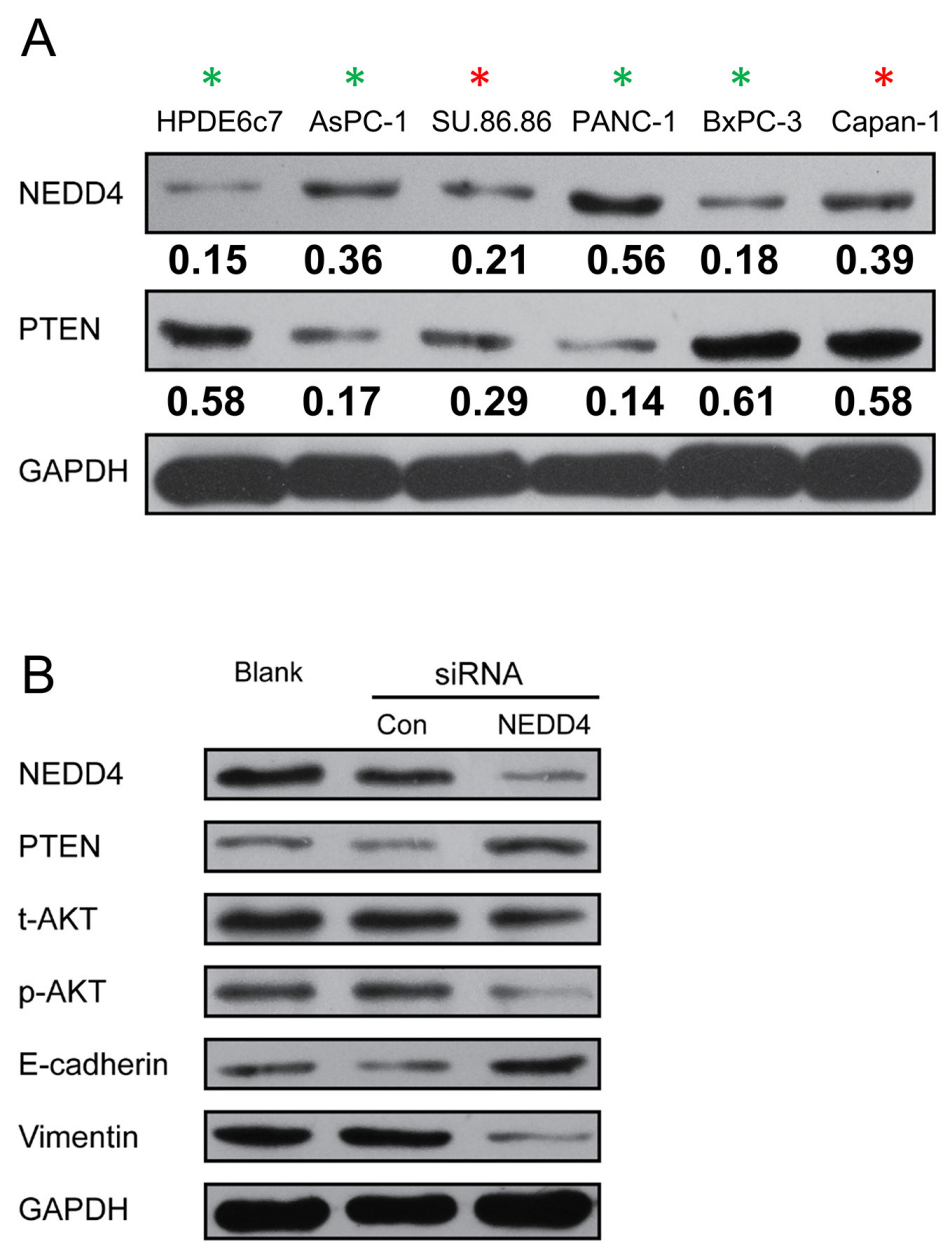
NEDD4 is heterogeneously expressed in PDAC cells and cross-talk with the PTEN/PI3K/AKT signaling pathway **A**. The expression of NEDD4 and PTEN in indicated PDAC cell lines, and the normal immortalized human pancreatic duct epithelial cell line, HPDE6c7. The numbers below the NEDD4 and PTEN blots show the relative quantification as obtained by the Image J-mediated densitometry analyses of the blots shown on the left, normalized to GAPDH expression. **B**. Western blot analysis demonstrating the effect of siRNA-related depletion of *NEDD4* on PI3K/Akt activation, PTEN expression, and epithelial (E-cadherin) and mesenchymal cell marker (vimentin) expression.

We first determined the steady state protein expression of NEDD4 and PTEN in the PDAC cell lines AsPC-1, BxPC-3, Capan-1, PANC-1, and SU.86.86, and a normal immortalized human pancreatic duct epithelial cell line, HPDE6c7. We observed that the expression of both proteins was highly heterogeneous in the PDAC cell lines, with highest NEDD4 expression noted in the PANC-1 cell line (Figure 1A). NEDD4 expression was higher in all the PDAC cell lines compared to the HPDE6c7 cell line.

Interestingly, NEDD4 was expressed at low levels and PTEN was expressed robustly in the normal immortalized human pancreatic duct epithelial cell line, HPDE6c7. Robust PTEN expression was also noted in the PDAC cell lines, BxPC-3 and Capan-1. Cumulatively, this indicated that either there was no correlation between NEDD4 and PTEN expression in the PDAC cell lines, or the correlation is context-dependent and hence not observed universally across all PDAC cell lines.

### Depletion of NEDD4 suppresses PTEN/PI3K/AKT signaling pathway in the PANC-1 cell line and restores an epithelial signature on the cells

The rational for choosing the PANC-1 cells for subsequent experiments was that it had the highest NEDD4 and lowest PTEN expression and hence provide an ideal scenario to test the functionality of *NEDD4* in PDAC. For our subsequent experiment, we transfected *NEDD4* siRNA in PANC-1 cells for 48 hours, which showed a significant reduction of NEDD4 protein amount compared to control cells (Figure 1B). It was suggested that NEDD4 is an oncoprotein since it targets PTEN for proteasomal degradation [10, 14–16]. Hence, we investigated the steady state levels of PTEN and PI3K/AKT signaling pathway members in the parental cells and those harboring the control or *NEED4* siRNA. NEDD4 depletion resulted in an increase in the expression of PTEN (Figure 1B). PI3K/AKT signaling pathway expression, which is negatively regulated by PTEN, was altered in our NEDD4 depleted cell lines (Figure 1B). The expression level of total AKT did not change, whereas the expression of phosphorylated AKT was decreased (Figure 1B) in *NEDD4* siRNA-transfectants. This suggests that *NEDD4* promotes PI3K/AKT signaling pathway and that PTEN can limit this activation.

**Figure 2:**
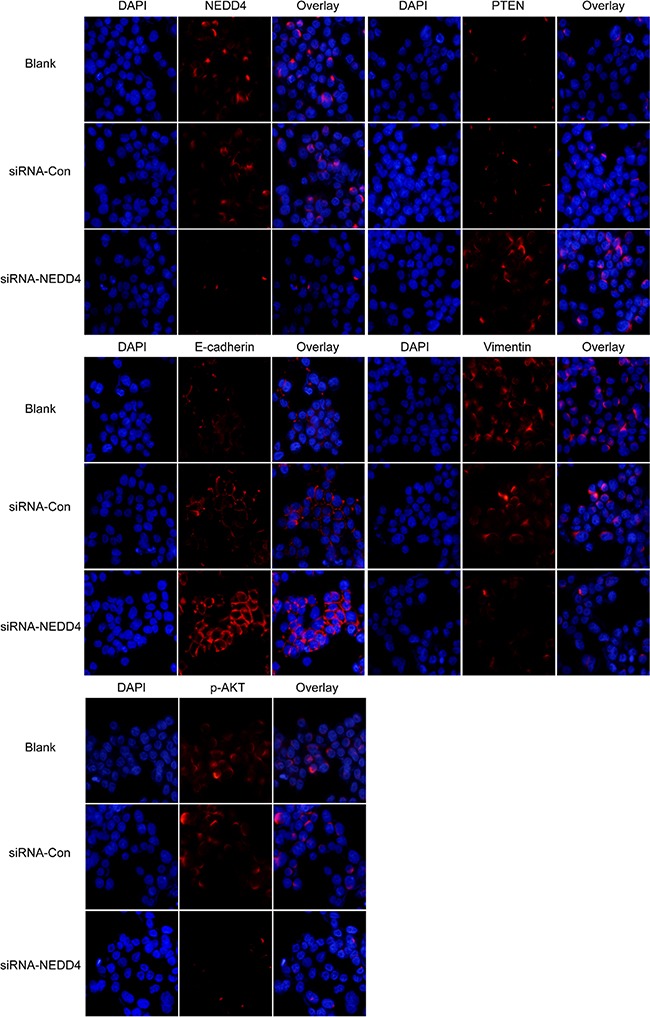
NEDD4 may affect PTEN/PI3K/AKT signaling pathways NEDD4, PTEN, p-AKT, E-cadherin and vimentin were detected by immunofluorescence. Depletion of *NEDD4* negatively affected PTEN expression. A decreased phosphorylation level of AKT associated with acquisition of E-cadherin and loss of vimentin (200x) was also observed.

We probed for the epithelial cell marker, E-cadherin, and the mesenchymal cell marker, vimentin, in *NEDD4* siRNA transfectants. *NEDD4* depleted PANC-1 cells resulted in a signature mimicking mesenchymal to epithelial cell transition (MET), with increased E-cadherin and decreased vimentin protein levels (Figure 1B, Figure 2).

### Depletion of NEDD4 suppresses the ability of in vitro growth, migration, and invasion in PDAC cells

**Figure 3:**
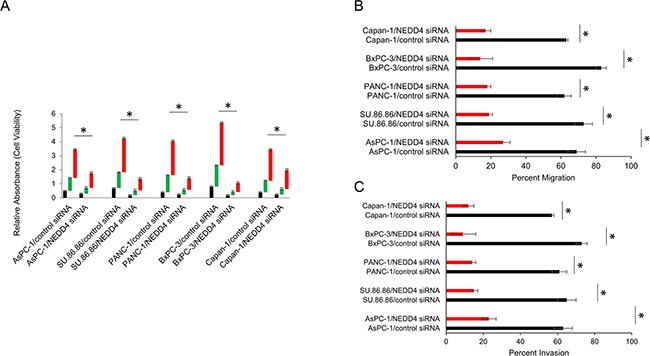
Depletion of *NEDD4* inhibited proliferation, migration and invasion in PDAC cells **A**. Cell proliferation was analyzed by MTT. Indicated cells were pretreated with *NEDD4* siRNA and proliferation was compared at 24, 48, and 72 hours. Depletion led to a significant inhibition of cell proliferation. **B, C**. Wound-healing and transwell invasion assays, respectively, revealed inhibitory effect on cell migration and invasion ability, similarly to proliferation inhibition experiment. *P<0.05.

It has been reported that NEDD4 might be an important player in malignant transformation processes [8, 9]. We thus investigated whether depletion of *NEDD4* would have an impact on PANC-1 cells ability to grow and to migrate. *NEDD4* silencing resulted in decreased proliferation in PANC-1 cells as assessed by the MTT assay (Figure 3A), suggesting that *NEDD4* potentiates cell proliferation. Wound-healing experiment results showed that *NEDD4* silencing impaired *in vitro* migration (Figure 3B) and invasion (Figure 3C) in the PANC-1 cells. Given the heterogeneous expression of NEDD4 observed in PDAC cell lines (Figure 1A) and to confirm the observed role of *NEDD4* expression in PANC-1 cells, we evaluated the effect of *NEDD4* silencing on cell proliferation and *in vitro* migration and invasion in AsPC-1, SU86.86, BxPC3, and Capan-1 cell lines. RNAi-mediated silencing of *NEDD4* resulted in attenuated proliferation (Figure 3A) and *in vitro* migration (Figure 3B) and invasion (Figure 3C) in all the PDAC cell lines tested, indicating the pro-oncogenic role of *NEDD4* in PDAC pathology.

### Transcriptional upregulation or post-translational-modification-mediated stabilization does not explain the differential NEDD4 protein expression

We then asked the mechanism behind observed differences of NEDD4 protein expression in the different pancreatic cancer cell lines and the normal non-transformed pancreatic cell line observed in Figure 1A.

**Figure 4:**
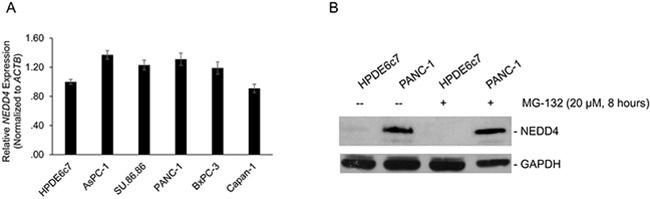
**A**. Quantitative real-time PCR (qRT-PCR)-based determination of change in steady-state *NEDD4* mRNA using total mRNA obtained from indicated cells. All panels are representative of a minimum of three experimental replicates. **B**. Inhibition of proteasome-mediated degradation results did not increase NEDD4 protein levels in the HPDE6c7 cells. Cells were incubated with MG-132 for 8 hours and immunoblotted for the indicated proteins. GAPDH served as a loading control.

Within the cell lines tested, the observed increase in relative NEDD4 protein expression (Figure 1A) was independent of changes in relative expression of steady state expression of *NEDD4* messenger RNA (mRNA) (Figure 4A). Furthermore, blocking proteasomal degradation by MG-132 treatment did not result in significant increase in steady-state levels of NEDD4 protein expression in the HPDE6c7 cells or the PANC-1 cells (Figure 4B). This suggested that the observed differential NEDD4 protein expression was not due to post-translational stabilization of the NEDD4 protein in the PANC-1 cell line.

### NEDD4 is translationally upregulated in PANC-1 cells

**Figure 5:**
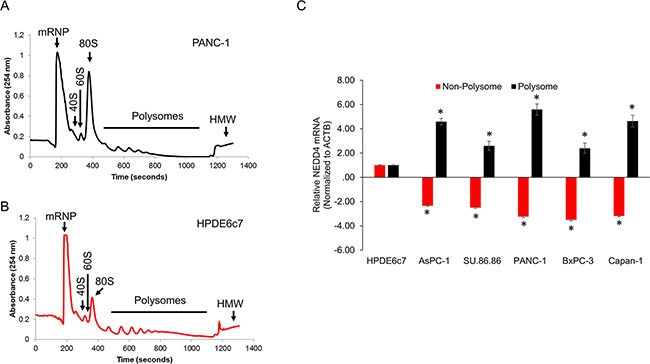
**A, B**. Representative polysome trace obtained by fractionation of HPDE6c7 (A) and PANC-1 (B) cells. **C**. QRT-PCR-based determination of change in polyribosomal bound *NEDD4* mRNA using non-polyribosomal and polyribosomal enriched mRNA obtained from the indicated cell lines. The data has been plotted relative to relative *NEDD4* mRNA in the HPDE6c7 cells. All panels are representative of a minimum of three experimental replicates. *P<0.05 compared to HPDE6c7 cells.

Since gene expression can be regulated at the level of mRNA transcription, translation, and post-translational modification and we ruled out two of them (Figure 4), we next set out to determine if the observed differences were due to differential translational regulation. We determined ribosomal occupancy of *NEDD4* mRNA in the HPDE6c7 and the PDAC cell lines. RNA isolated from the different polysome fractions (Figure 5A, 5B) were subjected to quantitative real-time polymerase chain reaction (qRT-PCR). There was no difference in global translation efficiency between HPDE6c7 and PANC-1 cells as can be seen by similar polysomal peaks obtained in the two cell lines. As shown in Figure 5C, *NEDD4* mRNA was significantly more polysomally enriched in the PDAC cell lines compared to the HPDE6c7 cells. In addition, the relative polysomal enrichment observed in each of the PDAC cell lines mimicked the relative protein NEDD4 protein expression observed in these cells (Figure 1A). The specificity of the finding was proved by the fact that sequestration into the non-polysomal fractions in HPDE6c7 was limited to the *NEDD4* transcript and the control *ACTB* had similar pattern of ribosomal occupancy in all the cell lines tested. Taken together, our data shows that the *NEDD4* mRNA is translationally activated in pancreatic cancer cells.

## DISCUSSION

Pancreatic cancer is a heterogeneous disease that widely differs in their pathological characteristics and clinical behavior. Almost 60-80% of patients with pancreatic cancer are metastatic at diagnosis [17]. The mortality is almost identical to the incidence throughout the world, which underlines the high case fatality rate of this aggressive disease. Thus it is imperative to have prognostic and diagnostic markers of human pancreatic cancer that would aid initial detection and also in clinical decision making about treatment modality.

Incidents of PDAC in Asia have been increasing in recent years. Despite intensive research in the diagnostics and therapeutic approaches, such as surgery, chemotherapy, radiotherapy, endocrine and immunotherapy, the clinical outcome and prognosis of PDAC patients remains poor. Expression of *NEDD4* was directly associated with cancer progression, particularly metastasis [18–22]. Thus, NEDD4 is proposed as a prognostic biomarker. However, the expression of *NEDD4* and its impact on cancer development in PDAC is not known.

NEDD4 is an HECT E3 ubiquitin ligase. It has been shown to participate in the ubiquitination of the tumor suppressor gene PTEN, which in turn suggest that NEDD4 might have a pro-oncogenic role [18–22] *NEDD4* was found to be overexpressed in colorectal cancers and promoted tumor cell growth [18]. Inhibition of *NEDD4* expression significantly suppressed proliferation of non-small cell lung carcinoma (NSCLC) cells and tumor growth *in vivo*. *NEDD4* over-expression augmented the tumorigenicity of lung cancer cells, while the *PTEN* gene product remained intact [20]. Similarly, it was shown that overexpression of *NEDD4* in colon cancer tissues was not correlated with down-regulation of *PTEN*. These results indicate that NEDD4 might be acting through PTEN-independent mechanisms. In fact, using conditional knockout mice it was shown that *Nedd4-1* is required for axonal growth in murine central nervous system neurons and that PTEN is not a substrate of Nedd4-1 ubiquitination in this context [23]. In fact, PTEN limited Nedd4-1 protein levels by modulating the activity of mTORC1, a protein complex that controls protein synthesis and cell growth [23]. Our results also suggest that in the context of PDAC, PTEN might not be a substrate of the NEDD4 ubiquitin ligase. Whether the suppression of *NEDD4* translation is dictated by PTEN-mediated effect of mTORC1-dependent protein synthesis need to be determined; however, given the heterogeneous expression of PTEN observed in the PDAC cell lines, and observation of high NEDD4 protein even in Capan-1 cells which had high PTEN expression, indicates that there are other potential mechanism for the *NEDD4* transcript-specific translational regulation observed in the PDAC cell lines.

In our study, we found that depletion of *NEDD4* suppressed the ability of growth and migration in PDAC cell lines. In other words, NEDD4 protein expression could possibly promote the carcinogenesis and cancer progression processes, such as PDAC cell proliferation, migration and invasion. The phosphorylation of AKT was suppressed. This indicates that NEDD4 influences pancreatic ductal adenocarcinoma progression and metastasis through PI3K/Akt signaling pathways. Our data for the first time show that NEDD4 promotes PDAC cell *in vitro* proliferation, migration, and invasion, which directly relates to tumorigenesis and disease progression. How NEDD4 mediates cell migration and invasion signaling is a crucial and perspective question and requires further investigation.

*NEDD4* has been shown to be transcriptionally upregulated by the Forkhead box protein M1B (FoxM1B) in human astrocytes [24, 25]. In pancreatic cancer cells it has been shown that the metastasis suppressor, N-myc downstream regulated gene-1 (*NDRG1*) inhibits NEDD4 expression [24]. Finally, Casein Kinase I (CKI)-mediated hyperphosphorylation of NEDD4 sensitizes the later for degradation by the SCFβ-TRCP ubiquitin E3 ligase complex [26]. Our experiments revealed a novel, translational regulatory mechanism for upregulation of translation of *NEDD4* mRNA. To the best of our knowledge, this is the first evidence of post transcriptional regulation of *NEDD4* expression. It is imperative that future experiments will have to focus on evaluating the existence of a similar post-transcriptional regulatory mechanism of *NEDD4* in patient samples and determining whether the aforementioned mechanisms dictating *NEDD4* expression in other contexts are also at interplay. The pleiotropic regulatory mechanisms determining NEDD4 protein expression point to the dynamic importance of inhibiting expression in normal epithelia, and it would not be surprising if multiple mechanisms redundantly regulated expression of *NEDD4* in normal pancreas and aberrantly during PDAC.

## MATERIALS AND METHODS

### Cell lines, growth conditions, and treatment

Human PDAC cell lines including AsPC-1, BxPC-3, Capan-1, PANC-1, and SU.86.86, and a normal immortalized human pancreatic duct epithelial cell line, HPDEE6c7, were obtained from American Type Culture Collection (ATCC, Manassas, VA, USA). All of the cells except for BxPC-3 and HPDE6c7 were cultured in DMEM containing 4.5 mg/mL d-glucose and l-glutamine (Life Technologies, Shanghai, China) supplemented with 10% FBS. BxPC-3 cells were grown in RPMI 1640 with 10% fetal bovine serum (FBS) (Lonza, Germany). HPDE6c7 cells were maintained in keratinocyte serum-free medium supplemented by epidermal growth factor and bovine pituitary extract (Life Technologies, Shanghai, China). Cells were kept at 37°C under a humidified atmosphere of 5% carbon dioxide. For the proteasomal inhibition, cells were treated with 20 μM of MG-132 (Sigma, Shanghai, China) for the indicated times.

### Quantitative real time PCR (qRT-PCR)

RNA was extracted from indicated cell lines using the Qiagen RNeasy mini kit, and the RT reaction was performed using the ABI TaqMan probes (Assay ID Hs00406454_m1 for *NEDD4* and Hs01060665_g1 for *ACTB*) as per manufacturer recommendations. Data was analyzed by the –ΔΔCt method and normalized to *ACTB* expression.

### Small interfering RNA and transfection

Indicated cells were transiently-transfected with *NEDD4*-targeting small interfering RNA (siRNA), sequence with 5′-UUCAAUUGCCAUCUGAAGUUUAUCC-3′ (Life Technologies, Shanghai, China). Non-targeting siRNA (Life Technologies, Shanghai, China) was used as control [26]. 5 × 10^5^ cells were seeded in 6-well plates and grown to 60-80% confluence. Transient transfection with *NEDD4* siRNA into PANC-1 cells using Lipofectamine 2000 (Life Technologies, Shanghai, China) for 72 hours. Simultaneously, non-targeting siRNA was transiently transfected at the same time. In all cases, successful silencing was verified by western blot (Figure 1B, and *data not shown*).

### Cell proliferation assay

Cell proliferation assay was performed via MTT assay (Sigma-Aldrich, Shanghai, China). 5 × 10^3^ cells were plated in 96-well plates and transfected with *NEDD4*-siRNA or non-targeting siRNA the following day. 20 μl MTT (5 mg/mL) were added to each well after 24, 48 and 72 hours, and incubated for 4 hours. 150 μl DMSO were subsequently added to each well and OD values were measured at 570 nm [27].

### Wound healing and invasion assays

*NEDD4*-siRNA was transfected into 4 × 10^5^ cells 24 hours post-seeding in 6-well plate. A wound was generated with a pipette tip. Dead cells were removed with PBS. An Olympus BX61 upright microscope was used to take the images of migration ability at 0 hours, 24 hours, 48 hours, and 72 hours. Migration distance was measured by Image J. Cell invasion assay was using transwell chamber assay as per manufacturer recommendations assay (Millipore, Beverly, MA, USA). To rule out compounding effect of *NEDD4*-siRNA on cell death and its resultant effect on *in vitro* migration and invasion, the cells in each case were treated with Mitomycin-C before starting each replicate of these experiments.

### Western blotting

Cells were harvested and lysed with RIPA lysis buffer containing protease and phosphatase inhibitor (Thermo Scientific, Beijing, China). Protein concentration was measured using the BCA-kit, and 20 μg of each sample was resolved by SDS-PAGE. The primary antibodies included rabbit anti-human NEDD4 (Abcam, ab14592, UK), mouse anti-human AKT, rabbit anti-human p-AKT (Santa Cruz Biotechnology, sc-5298/135650, Dallas, TX, USA), rabbit anti-human PTEN (Abcam, ab32199, UK); mouse anti-human E-cadherin (Abcam, ab1416, UK), mouse anti-human Vimentin (Abcam, ab8979, UK) and mouse anti-human glyceraldehyde 3-phosphate dehydrogenase (GAPDH) (Abcam, ab8245, UK). Optical densitometry analysis was performed using Image J software. GAPDH served as a loading control in each case.

### Polysome analysis

Polysome analysis was performed as described previously [28, 29]. RNA was extracted from different polysome fractions using Trizol LS reagent (Life Technologies, Shanghai, China) as per manufacturer recommendation. Quantitative real-time PCR was performed as described above.

### Immunocytochemistry (ICC)

Cells were grown on coverslips in 6-well dishes. Cells were transfected with *NEDD4*-siRNA for 48 hours and washed with PBS. Next, they were fixed with 2% (w/v) paraformaldehyde and permeabilized with 1% (v/v) Triton X-100. Afterwards, they were blocked with 10% (w/v) normal goat serum in phosphate-buffered saline (PBS) at room temperature for 1 hour. Finally, they were incubated in one of the primary antibodies overnight at 4°C. The following day, cells were washed and incubated for 1 hour with Cy3-labeled secondary antibody (Beyotime, Beijing, China) at room temperature, and then co-stained with DAPI (Sigma-Aldrich, Shanghai, China) to visualize the nuclei. Images were obtained using a fluorescence microscope at a magnification of 200x.

### Statistical analysis

Statistical analyses were conducted using SPSS software (version 17.0). All quantitative data were represented as mean ± standard deviation, determined from at least three independent experiments. Deviations were determined using Student's t test (2-tailed). Statistical evaluation of the data was performed with one-way ANOVA. *P* < 0.05 was considered as statistically significant.
